# Percutaneous Coronary Intervention in a Patient with Right-Sided Heart: Invert the Views?

**DOI:** 10.1155/2018/6073567

**Published:** 2018-03-07

**Authors:** Miguel López Hidalgo, Risshi D. Rampersad

**Affiliations:** ^1^Interventional Cardiology Unit, Hospital Metropolitano Del Norte, Valencia, Venezuela; ^2^Caribbean Heart Care, Medcorp Ltd., St Clair Medical Centre, Port of Spain, Trinidad and Tobago

## Abstract

Situs inversus totalis considered a malposition syndrome is a very rare condition, occurring in 1 : 8000 births, usually without associated structural congenital heart disease. The diagnosis is often incidental as in this case, which presented with an acute coronary syndrome as a manifestationof multivessel coronary artery disease. Here, we present the challenges encountered during the diagnosis and management of this entity via percutaneous intervention.

## 1. Introduction

Right-sided heart is a rare congenital anomaly that is related to malposition of the internal organs, and it can be found usually with all the organs changed in the opposite direction, known as situs inversus totalis (mirror-image dextrocardia) or less commonly as isolated dextrocardia in situs solitus (dextroversion) [1]. Mirror-image dextrocardia (situs inversus) has an incidence of associated congenital heart disease same as normal population, usually does not affect survival, and can be incidentally found in the presence of a cardiothoracic pathology such as coronary artery disease [2]. This situation offers a technical challenge to interventional cardiologists. Here, we describe a patient with postinfarction unstable angina having situs inversus with coronary artery disease, treated with percutaneous coronary intervention (PCI). The challenges in the diagnosis and treatment by an endovascular approach imposed by the anatomical changes of the usual topography of the heart and the presence of multivessel coronary artery disease as a very unusual presentation are shown and discussed.

## 2. Case Presentation

A 69-year-old Afro-American man was admitted in the emergency department of a public institution in Port of Spain, Trinidad, in February 2015 with EKG findings consistent of an acute coronary syndrome: an inferior wall ST elevation myocardial infarction (inferior STEMI). The symptoms of chest pain initiated 5 hours before admission, he was in Killip-Kimball class I, and he received thrombolytic therapy with Tenecteplase, with clinical, ECG, and biomarkers criteria of successful reperfusion and good clinical in-hospital outcome; primary PCI was not performed because there was not a catheterization laboratory available in that public hospital.

**Figure 1:**
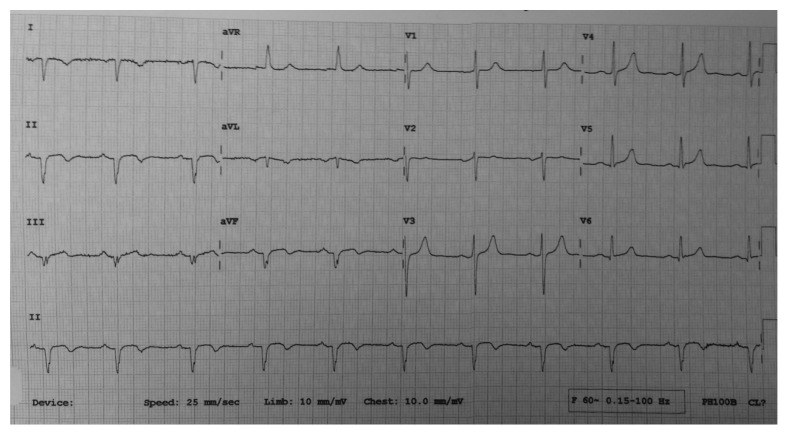
EKG shows the classical elements found in dextrocardia: right axis deviation of p wave with an inverted QRS axis of aVR. Signs of previous inferior MI: q waves in inferior leads.

The patient's prior history is that he smokes 10 to 15 cigarettes a day for the last 15 years and had no previous heart disease, hypertension, diabetes, and dyslipidemia nor family history of early coronary artery disease. He was discharged with medical treatment since he had an uncomplicated inferior myocardial infarction (MI) that was stratified for noninvasive evaluation, but due to the presence of postmyocardial infarction effort angina 5 days after discharge, he was admitted to Caribbean Heart Care Medcorp (CHCM) at St. Clair Medical Centre Hospital for diagnostic catheterization. The EKG revealed sinus rhythm with an inverted p wave in DI, aVL, and aVR, with right axis deviation, positive QRS complex in aVR, and poor progression of r waves in precordial leads and signs consistent of a prior inferior MI with q waves at DII, DIII, and aVF. The TIMI risk score was 2 (8% risk of event at 30 days). The EKG findings made us suspicious of a malposition syndrome (Figure 1).

**Figure 2:**
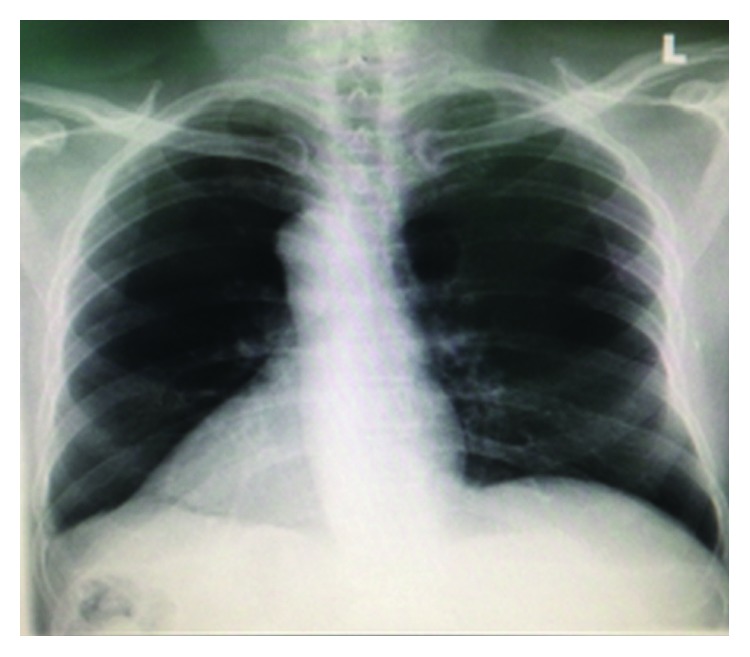
Chest radiogram shows the right-sided heart (apex to the right) and gastric chamber to the right, which adds to the diagnosis of situs inversus.

The physical exam with right-sided heart sounds, including a 4th heart sound and a right-sided apex with a palpable liver in the left side, and the radiogram findings with right-sided cardiac apex and gastric chamber Figure 2 confirmed our suspicion of situs inversus totalis, which was an incidental diagnosis.

Coronary angiography was performed via femoral access using the standard Judkins technique [3] with 6 French catheters (Judkins left: JL 4.0 and Judkins right: JR 3.5), which showed right-sided aortic arch and mirror image of coronary circulation. Horizontal axis mirror-image inversion of the angiographic views was used. A two-vessel coronary artery disease was diagnosed. There were a significant 70% stenosis at the proximal segment of the left anterior descending artery (LAD) and a right coronary artery (RCA) 90% stenosis in the distal segment.

**Figure 3:**
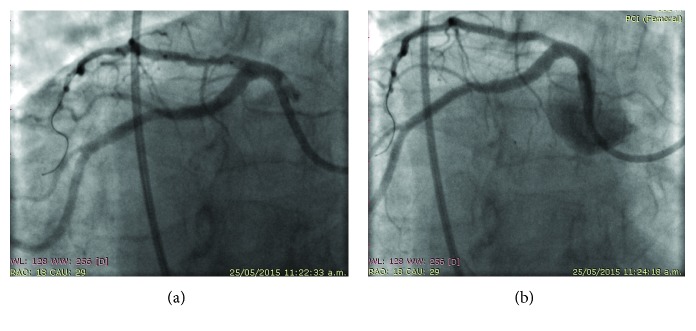
Sequence of percutaneous intervention with drug-eluting stent implantation in the proximal segment of the LAD. (a) Pre-PCI: shows a 70% stenosis at the proximal segment of LAD (stent in posistion, preimplantation). (b) Post-PCI: DES implantation.

**Figure 4:**
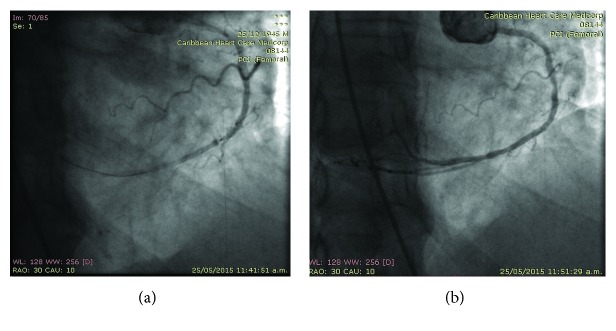
(a, b) Sequence of stent implantation in the distal segment stenosis of the right coronary artery with previous balloon predilatation.

On May 25, 2015, percutaneous coronary intervention (PCI) was performed with an extra-backup support EBU 3.5, 6 French guiding catheter with the standard Judkins technique, using inverted horizontal axis angiographic views of the left coronary artery, to mimic a left anterior oblique (LAO) with a cranial view for the left anterior descending artery (LAD), an inverted view; that is, to the other side, a right anterior oblique (RAO) view was used, to proceed with a direct stenting of the proximal segment stenosis of the LAD, implanting a 2.5 × 16 mm drug-eluting (everolimus) stent (Promus Premier, Boston Scientific) successfully (Figures 3(a) and 3(b)). Then, we proceeded to treat the right coronary artery 90% distal segment stenosis; for this, we used a right Judkins 3.5, 6F guiding catheter with horizontal mirror imaging, using a RAO view to mimic the LAO view; the anatomical right coronary artery was engaged easily using an opposite maneuver to the usual, with the tip of the catheter pointing leftward and rotated counterclockwise (instead of rotating clockwise), and then, we passed a Whisper 0.014 angioplasty guidewire and crossed the distal stenosis; predilatation was done with a 1.5 × 10 mm balloon (Apex, Boston Scientific), and then, a 2.5 × 12 mm zotarolimus-eluting stent (Resolute Integrity, Medtronic) was successfully implanted, obtaining excellent angiographic results and final TIMI grade 3 flow (Figures 4(a) and 4(b)).

The patient had an excellent in-hospital follow-up, without any symptoms and complications; there was no rise in cardiac biomarkers and was discharged after 48 hrs. With life style modification and receiving coated aspirin 81 mg/d, clopidogrel 75 mg/d, and atorvastatin 80 mg/d, a 3-month follow-up showed no recurrent ischemia, and a treadmill stress test (positioning electrodes to the right side) was performed which was negative.

## 3. Discussion

Dextrocardia is a rare cardiac anomaly of development, characterized by an opposite orientation of the apex to the usual one; it is in the right hemithorax, with its axis directed to the right and caudally. This can occur with situs solitus or more frequently in association with situs viscerum inversus totalis (all the organs inverted); it can also be found as situs ambiguous. With situs solitus and situs inversus, the atrial situs always corresponds to the visceral situs; with situs ambiguous, the arrangement of the organs is not as ordered, and the relation between the atria and the viscera is inconsistent [1, 2, 4]. Dextrocardia with situs inversus is an infrequent condition occurring with an estimated range of 1 : 8000 to 1 : 10,000. In situs inversus totalis, the incidence of structural congenital heart disease is low, estimated to be near 3%. It is known that dextrocardia with situs inversus has a 15% association of another rare disease that affects primarily the respiratory tract and the epithelia of the sexual organs, the Kartagener syndrome (immotile cilia syndrome characterized by bronchitis, chronic sinusitis, and infertility in men). It is believed that these patients share the same risk as the general population to develop ischemic heart disease because of the high prevalence of atherosclerosis [4]. The majority of patients with dextrocardia and situs inversus totalis are ignorant of their variant anatomy until they are forced to seek medical attention, most frequently for cardiothoracic pathology [1, 4]. Our patient was not aware of his malposition syndrome until this admission, when he presented for the first time with acute coronary syndrome. The presence of coronary artery disease (CAD) in this population presents several challenges to the interventional cardiologists due to the abnormal anatomic position of the heart, usually right position of the aortic arch and unfamiliar coronary anatomy especially at their origin. Once the diagnosis of dextrocardia is made in the context of an acute coronary syndrome, a reversed or right-sided ECG should be done; this will show the exact localization of the infarct in an ST elevation myocardial infarction, particularly if it is in the anterior and/or lateral wall [5].

The first report of diagnostic cardiac catheterization in a patient with dextrocardia was done in 1973 by Hynes. Fourteen years later, Moreyra and coworkers in 1987 in the USA performed the first balloon angioplasty in a patient with dextrocardia and single-vessel CAD [6], and PCI with balloon angioplasty in a dextrocardia patient with multivessel CAD has been reported from Japan by Yoshimasa Yabe since 1995 [7, 8]. The first stent deployment in a patient with dextrocardia in situs inversus was performed in Italy by Patti et al. in 1999 [9].

This is the first case of diagnostic and therapeutic catheterization of dextrocardia in situs inversus totalis documented in Trinidad. It was our first encounter with this rare scenario, so we had to search and review the literature to understand and approach this variety of presentation. We learned that, with the inversion technique of mirror-image views, it was easier to approach the lesions and that, with the RCA, the inverted maneuver of counterclockwise rotation as suggested by Gaglani et al. in 1989 [10] made the difference in engaging it. Note that with the image inversion technique, one gets a partly normalized intended view in the horizontal axis, but the apex is always pointing to the opposite side of the conventional angiogram; that is, to the right, to correct this, the double inversion technique was developed.

Although we only used the inversion technique to facilitate the viewing, this does not normalize completely the views. There have been some advances applied to the newer fluoroscopy equipment such as the “horizontal sweep reverse” feature used in the method of double-inversion technique for coronary angiogram viewing in dextrocardia, developed by Goel. With this, the unfamiliar angulated coronary angiograms seen in dextrocardia are digitally corrected into a conventionally normal angiogram [11–12].

In dextrocardia patients, there are several considerations that should be taken into account: (1) image acquisition, a mirror image between LAO and RAO; using an image reversal technology will give an advantage to the operator for the intervention; (2) the technique of torque of the catheter, a reverse maneuver of the usual between clockwise and counterclockwise rotation for the RCA; (3) transfemoral access gives a better access, even though some reports of transradial approach are also good for dextrocardia cases; and (4) anatomical variation in dextrocardia, variable position of the aortic arch (left/right), will require different catheter selection, that is, in the presence of a right aortic arch, as in this case, but in a case with an isolated right aortic arch, a left Judkins catheter can be used to selectively cannulate the left coronary artery and is also able to cannulate a right-sided right coronary artery [9–12].
